# Comparison of longitudinal changes in four surrogate insulin resistance indexes for incident T2DM in middle-aged and elderly Chinese

**DOI:** 10.3389/fpubh.2022.1046223

**Published:** 2022-11-30

**Authors:** Liang Pan, Yu Gao, Jing Han, Ling Li, Miyuan Wang, Hongye Peng, Juan Liao, Hua Wan, Guohua Xiang, Yangyun Han

**Affiliations:** ^1^Phase 1 Clinical Trial Center, Deyang People's Hospital, Sichuan, China; ^2^College of Chinese Medicine, Beijing University of Chinese Medicine, Beijing, China; ^3^The First College of Clinical Medical Science, China Three Gorges University, Yichang, China; ^4^Division of Central Archives, Deyang People's Hospital, Sichuan, China; ^5^School of Public Health, Huazhong University of Science and Technology, Wuhan, China; ^6^Graduate School, Beijing University of Chinese Medicine, Beijing, China; ^7^Department of Science and Education, Deyang People's Hospital, Sichuan, China; ^8^Deyang Maternal and Child Health Service Center, Sichuan, China; ^9^Deyang People's Hospital, Sichuan, China

**Keywords:** type 2 diabetes mellitus, longitudinal changes, triglyceride glucose index, Chinese visceral adiposity index, lipid accumulation product, triglyceride/high-density lipoprotein cholesterol, area under curve, surrogate index

## Abstract

**Aims:**

Previous studies suggested a significant relationship between four surrogate indexes of insulin resistance and subsequent type 2 diabetes mellitus (T2DM). But the association of longitudinal changes (denoted as *-D*) in CVAI (Chinese visceral adiposity index), LAP (lipid accumulation product), TyG (triglyceride-glucose), and TG/HDL-C (triglyceride/ high-density lipoprotein cholesterol) indexes with the risk of T2DM remained uncertain. We aimed to compare the changes in those four surrogate indexes for predicting T2DM in middle-aged and elderly Chinese.

**Methods:**

We extracted data from the China Health and Retirement Longitudinal Study (CHARLS). Multivariate logistic regression models were used to estimate odds ratio (OR) with 95% confidence interval (CI) of incident T2DM with four surrogate indexes. The restricted cubic spline analysis was used to examine potential non-linear correlation and visualize the dose-response relationship between four indexes and T2DM. The receiver operator characteristic curve was used to compare the performance of the four indexes to predict T2DM.

**Results:**

We enrolled 4,596 participants in total, including 504 (10.97%) with T2DM. Analysis results showed that four surrogate indexes were associated with T2DM, and the multivariate-adjusted ORs (95% CIs) of T2DM were 1.08 (1.00–1.16), 1.47 (1.32-1.63), 1.12 (1.00–1.25), and 2.45 (2.12–2.83) for each IQR (interquartile range) increment in CVAI-D, LAP-D, TG/HDLC-D, and TyG-D, respectively. Restricted cubic spline regression showed a non-linear correlation between four surrogate indexes and the risk of T2DM (*p* for non-linear < 0.001). From the ROC (receiver operating characteristic) curve, TyG-D had the highest AUC (area under curve), and its AUC values were significantly different from other three indexes both in male and female (all *P* < 0.001).

**Conclusion:**

Compared with other indexes, TyG-D was a better predictor in the clinical setting for identifying middle-aged and elderly Chinese with T2DM. Monitoring long-term changes in TyG might help in the early identification of individuals at high risk of T2DM.

## Introduction

The prevalence of type 2 diabetes mellitus (T2DM) is a growing global health issue (1, 2). Presently, about 1 in 11 adults have diabetes mellitus (DM), 90% of whom develop T2DM (3). Asia is a significant region of the fast spreading T2DM global epidemic. During the past few decades, the prevalence of T2DM has increased significantly in China (4).

T2DM is known as a complex and heterogeneous disease, for which obesity is one of the strongest risk factors (5). Increased (mostly visceral) adipose tissue mass-related insulin resistance (IR) has been recognized as a critical component that may contribute to concurrent increases in the prevalence of T2DM (6). IR is present in many metabolic disorders, such as T2DM and metabolic syndrome (7, 8). However, due to its complicated process, lengthy laboratory test, and high cost, the method for the IR measurement is not widely used in clinical trials or sizable population-based studies (9). Excessive abdominal fat accumulation has been linked to glucose and lipid metabolic problems (10). In particular, increased visceral adipose tissue (VAT) has been linked to IR (11). In diabetic studies, the Chinese visceral adiposity index (CVAI) and lipid accumulation product (LAP) have been employed as trustworthy indicators of visceral adiposity (12–14). Triglyceride-glucose (TyG) index and the triglyceride-to-HDL-cholesterol (TG/HDL-C) ratio have also emerged as straightforward, affordable, repeatable, and reliable surrogates for IR measurement (15).

Previous studies show that high TyG index is a risk factor for incident DM and cardiovascular disease (16, 17). However, a drawback of those studies is that they only examine data from one moment in time and do not examine how the index changes over time. Given that each individual has unique underlying conditions, assessing dynamic changes may be more beneficial than measuring data at a single time point (18). We calculate the difference in index value at the end of follow-up (2015) minus that at baseline (2011) for analysis, denoted as *-D*. This research aims to assess the applicability of longitudinal changes in CVAI, LAP, TyG, and TG/HDL-C index in predicting T2DM in middle-aged and elderly Chinese population in a large community-based prospective cohort study.

## Methods

### Study population

Our study obtained data from 2011 wave and 2015 wave of the CHARLS survey completed by the National School of Development at Peking University, which was open to the public (http://charls.pku.edu.cn/). CHARLS was a large-scale, multidisciplinary survey conducted for nation-wide residents aged 45 and older in 450 villages and communities across 28 provinces (autonomous regions and municipalities). It collected data on a wide range of topics, including basic demographics, family, health status and function, cognition and depression, health care and insurance, work and retirement, pensions, income expenditure and assets, and housing. It is a trustworthy resource for researching middle-aged and elder people's health statuses and contributing elements. By the year 2015, 12,241 households with a total of 21,097 residents were represented in the survey.

Professional staff took venous blood samples from participants in 2011 and 2015 after overnight fasting for at least 12 h. Complete blood count was performed right away at the survey sites. The full blood samples were kept at 4°C while the other samples were delivered to the central laboratory in Beijing (Youanmen Centre for Clinical Laboratory of Capital Medical University) for further testing. An enzymatic-colourimetric test was used to determine the levels of glucose, total cholesterol (TC), triglyceride (TG), low-density lipoprotein cholesterol (LDL-C), and high-density lipoprotein cholesterol (HDL-C). The levels of glycated hemoglobin (HbA1c) were measured using high-performance liquid chromatography with boronate affinity.

The initial approval of CHARLS was granted by Peking University's Ethical Review Committee in 2008 (IRB00001052–11,015). The research methods followed all applicable CHARLS requirements and guidelines. Before participating willingly in CHARLS, each individual completed informed consent forms.

We finalized the inclusion criteria for the participants in this study: 45 years and older; complete demographic data including gender, level of education, marital status, and residential location; complete fasting blood glucose levels. Based on those inclusion criteria, a total of 4,596 participants from 17,708 individuals were included.

### Measurement

#### Assessment of four surrogate indexes of insulin resistance

##### CVAI

Male: CVAI = −267.93 + 0.68 × age + 0.03 × BMI + 4.00 × WC (cm) + 22.00 × log10(TG) (mmol/L)– 16.32 × HDL-C (mmol/L)

Female: CVAI = −187.32 + 1.71 × age + 4.23 × BMI + 1.12 (cm) × WC + 39.76 × log10(TG) (mmol/L) – 11.66 × HDL-C (mmol/L) (19).

##### LAP

Male: LAP = [(WC (cm) – 65) × TG (mmol/L)]

Female: LAP = [(WC (cm) – 58) × TG (mmol/L)] (20).

##### TyG

Ln [fasting triglycerides (mg/dL) × fasting glucose (mg/dL)/2] (21).

##### TG/HDL-C

Ratio of serum triglycerides to high-density lipoprotein cholesterol (22).

##### CVAI-D, LAP-D, TyG-D, TG/HDLC-D

Changes in CVAI, LAP, TyG, TG/HDL-C indexes were calculated with their levels measured in 2015 minus that in 2011 (baseline).

#### Assessment of T2DM

We defined T2DM in Accordance with the 2005 American Diabetes Association criteria: a HbA1c level of 6.5 percent or higher; a fasting blood glucose level of 126 mg/dL (7 mmol/L) or higher; a random blood glucose level of 200 mg/dL (11.1 mmol/L) or higher, and/or self-reported diagnosis (“Have you ever been diagnosed with diabetes or hyperglycemia?”).

The analyses were adjusted for anthropometric data, health-related activities, and socio-demographic factors. Age, gender, education (primary school or below, high school, college or above), location (city/town, village), and marital status (married, non-married), were all analyzed as demographic variables. As for factors of health-related behavior, we analyzed smoking status (“non-smoker,” “ex-smoker,” “current smoker”), drinking status (“never”, “less than once a month”, “more than once a month”), and sleep time. Those data came from self-reported questionnaires and were gathered with the aid of skilled interviewers. Athropometric data such as systolic blood pressure (SBP) and diastolic blood pressure (DBP) were the means calculated from three measurements using Omron HEM-7200 sphygmomanometer.

### Statistical analysis

Classified variables were represented by percentages, while continuous variables were represented by means and standard deviation (SD) for normally distributed data and median (interquartile range, IQR) for non-normally distributed data. Based on the quartiles of four surrogate indexes (Q1, Q2, Q3, Q4), baseline characteristics and diabetes incident rate were compared using the One-Way ANOVA, Kruskal-Wallis H test or chi-square test, if necessary. In order to calculate the odds ratio (OR) of diabetes with a 95% confidence interval (CI) for four surrogate indexes as continuous (per IQR increment) or categorical (quartiles) variables, we employed logistic models. Three models were used to explore the relationship between four surrogate indexes and T2DM, including unadjusted crude model (Model 1); model adjusted for age, gender, education level, location and marital status (Model 2); model further adjusted for smoking status, drinking status and sleep time (Model 3). The results were presented with ORs and 95% CIs. The restricted cubic spline analysis was also performed to examine possible non-linear correlation and visualize the dose–response relationship between four surrogate indexes and T2DM. Additionally, to assess the predictive performance of LAP-D, TyG-D, CVAI-D, and TG/HDLC-D for T2DM, The area under the receiver operating characteristic (ROC) curve (AUC) value were determined. DeLong's non-parametric method was used to compare the AUC between TyG-D and other indexes (23). The optimal cutoff values of LAP-D, TyG-D, CVAI-D, and TG/HDLC-D for predicting T2DM were identified based on the maximum value of the youden idnex.

All statistical analyses were performed using R 4.1.3. The package “rms” was used for analyzing with restricted cubic splines. MedCalc version 13.0 for Windows (MedCalc Software, Mariakerke, Belgium) was used to perform significance tests for the comparison of AUCs. *P* < 0.05 for a two-tailed test denoted statistical significance.

## Results

### Baseline characteristics

Table 1 shows the characteristics of the study population. A total of 4,596 participants were included [median age = 58, including 2,152 (46.82%) male and 2,444 (53.18%) female]. Of all those included participants, 504 (10.97%) had T2DM while the rest had not. The baseline median of CVAI-D, LAP-D, TG/HDLC-D, and TyG-D in all participants was 7.46 (−7.08, 21.39), 17.37 (−23.42, 71.65), 0.18 (−0.51, 0.86), and 0.07 (−0.24, 0.38), respectively. Characteristics of participants with T2DM were obviously different from those without, specifically, the formers were more likely to be older, and with higher SBP, DBP, BMI, WC, Glucose, TG, CVAI-D, LAP-D, TG/HDLC-D, TyG-D and lower HDL-C.

**Table 1:** The characteristics of the study participants grouped by T2DM at baseline (*N* = 4,596).

	**Total**	**Non-T2DM**	**T2DM**	***p***
	**(*n* = 4,596)**	**(*n* = 4,092)**	**(*n* = 504)**	
Age	58.00 (52.00, 65.00)	58.00 (52.00, 65.00)	60.00 (53.00, 67.00)	<0.01
**Gender**				0.67
Female	2,444 (53.18)	2,181 (53.30)	263 (52.18)	
Male	2,152 (46.82)	1,911 (46.70)	241 (47.82)	
**Marita**				0.38
Non-married	516 (11.23)	453 (11.07)	63 (12.50)	
Married	4,080 (88.77)	3,639 (88.93)	441 (87.50)	
**Education**				0.39
Primary school or below	3,267 (71.08)	2,897 (70.8)	370 (73.41)	
High school	929 (20.21)	832 (20.33)	97 (19.25)	
College or above	400 (8.70)	363 (8.87)	37 (7.34)	
**Location**				0.67
City/Town	4,335 (94.32)	3,857 (94.26)	478 (94.84)	
Village	261 (5.68)	235 (5.74)	26 (5.16)	
**Smoking**				0.51
Non-smoker	2,803 (60.99)	2,507 (61.27)	296 (58.73)	
Current smoker	1,432 (31.16)	1,264 (30.89)	168 (33.33)	
Ex-smoker	361 (7.85)	321 (7.84)	40 (7.94)	
**Drinking**				0.13
Never	3,056 (66.49)	2,706 (66.13)	350 (69.44)	
Less than once a month	371 (8.07)	341 (8.33)	30 (5.95)	
More than once a month	1,169 (25.44)	1,045 (25.54)	124 (24.60)	
Sleep time	6.25 (5.00, 8.00)	6.50 (5.00, 8.00)	6.00 (5.00, 8.00)	0.91
BMI (kg/m^2^)	22.83 (20.71, 25.28)	22.7 0 (20.64, 25.09)	23.80 (21.18, 26.61)	<0.01
WC (cm)	83.50 (77.00, 90.03)	83.00 (77.00, 90.00)	87.00 (79.00, 94.00)	<0.01
Glucose (mg/dl)	99.90 (93.24, 106.92)	99.36 (92.88, 106.20)	104.76 (96.66, 111.78)	<0.01
TG (mg/dl)	99.12 (71.68, 138.06)	97.35 (70.8, 136.29)	107.97 (80.54, 151.78)	<0.01
HDL-C (mg/dl)	51.03 (42.91, 61.47)	51.42 (43.3, 61.47)	48.33 (39.82, 59.63)	<0.01
SBP (mmHg)	125.33 (113.33, 139.67)	124.67 (112.67, 139.08)	130.00 (119.33, 143.67)	<0.01
DBP (mmHg)	74.00 (66.67, 82.33)	73.67 (66.33, 82.00)	76.33 (69.67, 85.00)	<0.01
CVAI-D	7.46 (−7.08, 21.39)	6.98 (−7.34, 20.95)	10.83 (−4.15, 25.87)	<0.01
LAP-D	17.37 (−23.42, 71.65)	15.19 (−24.76, 66.66)	41.16 (−11.95, 121.52)	<0.01
TG/HDLC-D	0.18 (−0.51, 0.86)	0.18 (−0.5, 0.83)	0.26 (−0.56, 1.26)	0.05
TyG-D	0.07 (−0.24, 0.38)	0.04 (−0.26, 0.35)	0.33 (0.02, 0.69)	<0.01

*p*-values were calculated from chi-square tests (categorical variables) or rank-sum tests (non-normally distributed continuous variables) or *t*-tests (normally distributed continuous variables).

T2DM, type 2 diabetes mellitus; BMI, body mass index; WC, waist circumference; DBP, diastolic blood pressure; SBP, systolic blood pressure; TG, triglyceride; HDL-C, high-density lipoprotein; CVAI-D, difference in Chinese visceral adiposity index; LAP-D, difference in lipid accumulation product; TG/HDLC-D, difference in triglyceride/high-density lipoprotein cholesterol ratio; TyG-D, difference in triglycerides/ glucose index.

### Association and dose-response relationship between four surrogate indexes and T2DM

Table 2 shows the association between four surrogate indexes and the risk of T2DM, as well as their quartiles. Multivariable logistic regression analysis was performed after adjusting for age, gender, education level, location, marital status, smoking status, drinking status, sleep time, SBP and DBP. Results showed that four surrogate indexes were associated with T2DM as continuous variables. Specifically, each IQR increment in CVAI-D was associated with 8% higher odds for T2DM (OR, 1.08; 95% CI, 1.00–1.16); each IQR increment in LAP-D was associated with 47% higher odds for T2DM (OR, 1.47; 95% CI, 1.32–1.63); each IQR increment in TG/HDLC-D was associated with 12% higher odds for T2DM (OR, 1.12; 95% CI, 1.00–1.25); each IQR increment in TyG-D was associated with 145% higher odds for T2DM (OR, 2.45; 95% CI, 2.12–2.83). The risk of T2DM increased gradually with the quartiles of the CVAI-D, LAP-D, AND TyG-D (*P* for trend <0.001). Compared to the first quartile group (Q1), the last quartile group (Q4) in CVAI-D,LAP-D, TG/HDLC-D, and TyG-D presented the highest risk of T2DM (OR = 1.63, 95% CI = 1.25–2.12; OR = 2.24, 95% CI = 1.73–2.90; OR = 1.33, 95% CI = 1.04–1.71; OR = 3.72, 95% CI = 2.83–4.93, respectively).

**Table 2:** Association between four surrogate indexes and T2DM risk.

	**Model 1**	***P***	**Model 2**	***P***	**Model 3**	***P***
CVAI-D per IQR	1.06 (0.99, 1.14)	0.095	1.07 (1.00, 1.15)	0.048	1.08 (1.00, 1.16)	0.04
**Quartiles of CVAI-D**
Q1	Reference	——	Reference	——	Reference	——
Q2	1.00 (0.76, 1.32)	1	1.03 (0.77, 1.36)	0.853	1.02 (0.77, 1.36)	0.869
Q3	1.21 (0.92, 1.58)	0.171	1.26 (0.96, 1.65)	0.102	1.27 (0.96, 1.67)	0.09
Q4	1.21 (0.92, 1.58)	0.002	1.58 (1.22, 2.06)	0.001	1.63 (1.25, 2.12)	<0.001
*P* for trend		<0.001		<0.001		<0.001
LAP-D per IQR	1.44 (1.30, 1.60)	<0.001	1.49 (1.34, 1.65)	<0.001	1.47 (1.32, 1.63)	<0.001
**Quartiles of LAP-D**
Q1	Reference	——	Reference	——	Reference	——
Q2	0.99 (0.74, 1.32)	0.941	0.96 (0.72, 1.29)	0.804	0.98 (0.73, 1.31)	0.905
Q3	1.00 (0.75, 1.33)	1	1.02 (0.76, 1.36)	0.901	1.03 (0.77, 1.38)	0.844
Q4	2.15 (1.67, 2.78)	<0.001	2.28 (1.77, 2.95)	<0.001	2.24 (1.73, 2.90)	<0.001
*P* for trend		<0.001		<0.001		<0.001
TG/HDLC-D per IQR	1.11 (0.99, 1.24)	0.056	1.12 (1.00, 1.25)	0.047	1.12 (1.00, 1.25)	0.047
**Quartiles of TG/HDLC-D**
Q1	Reference	——	Reference	——	Reference	——
Q2	0.87 (0.67, 1.14)	0.311	0.85 (0.65, 1.10)	0.219	0.86 (0.65, 1.12)	0.255
Q3	0.70 (0.53, 0.92)	0.012	0.69 (0.52, 0.91)	0.009	0.70 (0.53, 0.92)	0.012
Q4	1.31 (1.03, 1.68)	0.029	1.33 (1.04, 1.71)	0.023	1.33 (1.04, 1.71)	0.024
*P* for trend		0.076		0.057		0.06
TyG-D per IQR	2.38 (2.06, 2.74)	<0.001	2.45 (2.12, 2.83)	<0.001	2.45 (2.12, 2.83)	<0.001
**Quartiles of TyG-D**
Q1	Reference	——	Reference	——	Reference	——
Q2	0.91 (0.65, 1.28)	0.606	0.91 (0.65, 1.28)	0.594	0.90 (0.64, 1.27)	0.56
Q3	1.83 (1.36, 2.47)	<0.001	1.85 (1.37, 2.50)	<0.001	1.79 (1.33, 2.42)	<0.001
Q4	3.58 (2.74, 4.75)	<0.001	3.71 (2.83, 4.92)	<0.001	3.72 (2.83, 4.93)	<0.001
*P* for trend		<0.001		<0.001		<0.001

Model 1 was crude model; Model 2 adjusted for age, gender, education level, location and marital status; Model 3 was further adjusted for smoking status, drinking status and sleep time.

IQR, interquartile range; CVAI-D, difference in Chinese visceral adiposity index; LAP-D, difference in lipid accumulation product; TG/HDLC-D, difference in triglyceride/high-density lipoprotein cholesterol ratio; TyG-D, difference in triglycerides/glucose index.

**Figure 1:**
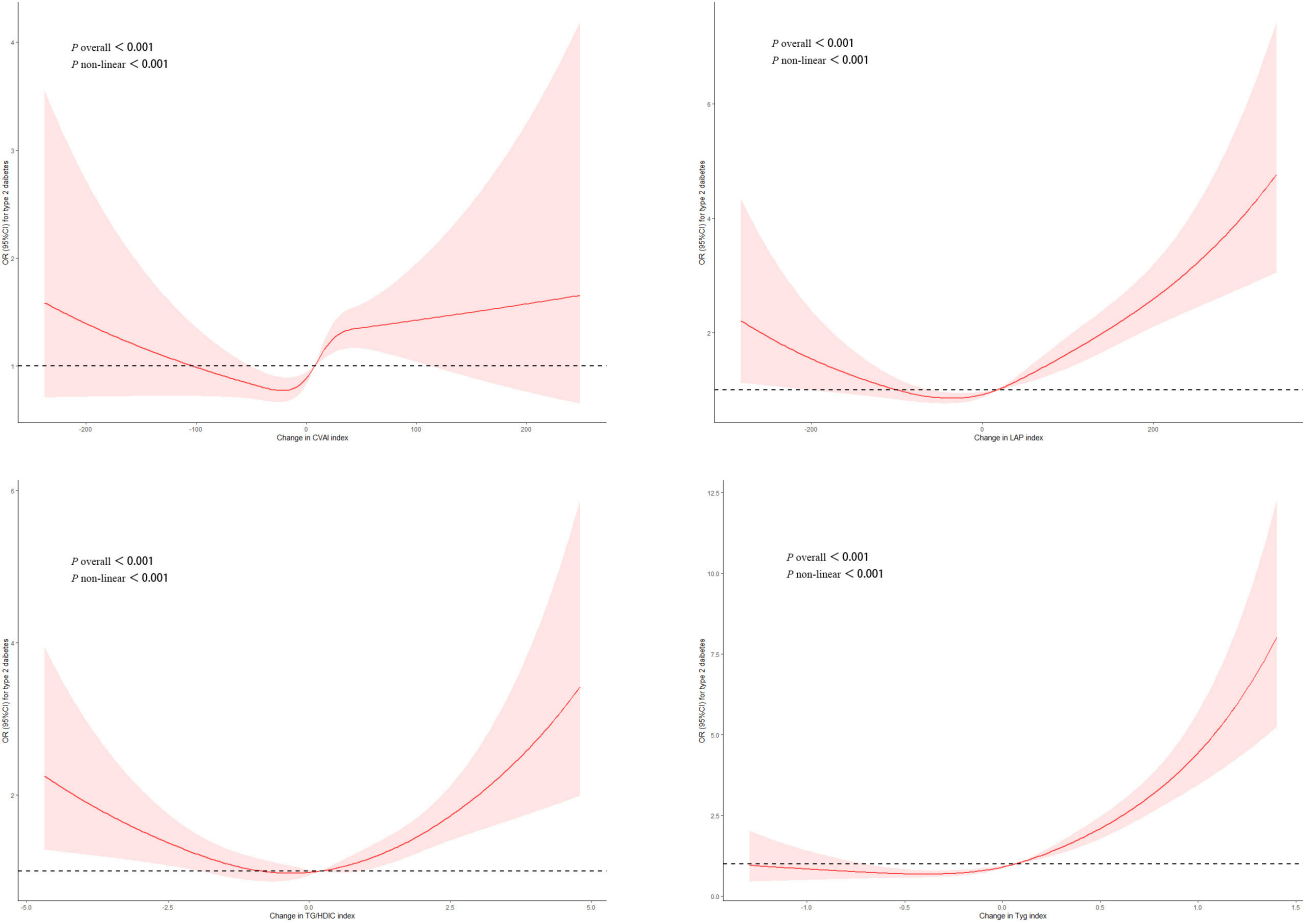
Adjusted cubic spline model of the relationship between four surrogate indexes and T2DM risk. A *U*-shaped association between four surrogate IR indexes and the risk of T2DM.

Figure 1 shows the dose-response relationship between four surrogate indexes and the risk of T2DM. Restricted cubic spline regression showed a non-linear dose-response relationship between four surrogate indexes and the risk of T2DM (*P*_overall_ < 0.001, *P*_non − liner_ < 0.001). And we found that there was U-shaped association between four surrogate indexes and the risk of T2DM.

### Predictive performance of the four surrogate indexes for T2DM by gender

**Figure 2:**
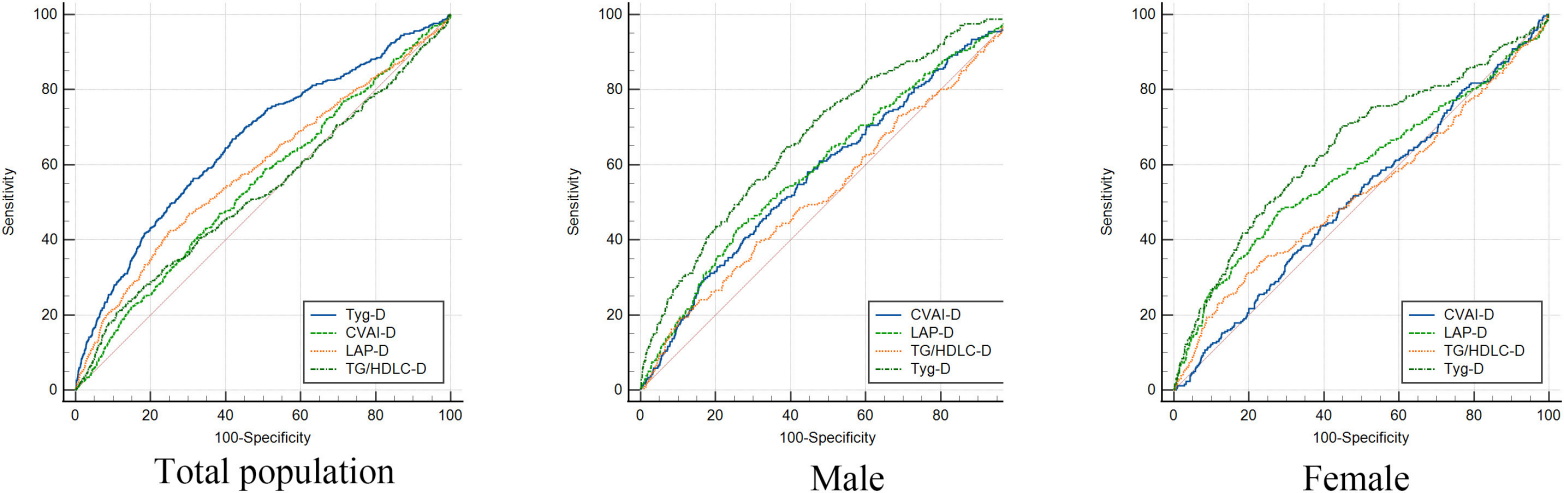
Receiver operating characteristic curves of four surrogate indexes in total population, male and female for incident T2DM. TyG-D presented the highest diagnostic value of all.

Table 3 shows the predictive performance of four surrogate indexes for T2DM risk. TyG-D had the highest AUC [0.66 (0.63–0.69)], followed by LAP-D [0.59 (0.56–0.62)], CVAI-D [0.55 (0.52–0.57)], and TG/HDLC-D [0.53 (0.50–0.56)]. TyG-D had the highest sensitivity (0.669) and TG/HDLC-D had the highest specificity (0.890) of all. Gender-stratified analyses showed that TyG-D had the highest diagnostic value of all (Figure 2). Among the female participants, TyG-D had the highest AUC [0.65 (0.61–0.69)], followed by LAP-D [0.59 (0.55–0.63)], TG/HDLC-D [0.53 (0.49–0.57)], and CVAI-D [0.51 (0.48–0.55)]. Among the male participants, TyG-D also had the highest AUC [0.68 (0.64–0.71)], followed by LAP-D [0.60 (0.56–0.64)], CVAI-D [0.58 (0.54–0.62)], and TG/HDLC-D [0.53 (0.49–0.57)].

**Table 3:** Comparison of predictive accuracy and cut-off values of four surrogate indexes for T2DM by gender.

	**Test**	**AUC**	**95 CI low**	**95 CI up**	**Cutoff value**	**Specificity**	**Sensitivity**	**PPV**	**NPV**	***P***
Female	CVAI-D	0.51	0.48	0.55	13.05	60.89	43.73	0.12	0.90	0.484
	LAP-D	0.59	0.55	0.63	70.34	72.31	47.53	0.17	0.92	<0.001
	TG/HDLC-D	0.53	0.49	0.57	1.10	80.61	31.18	0.17	0.90	0.223
	TYG-D	0.65	0.61	0.69	0.12	55.66	69.58	0.16	0.94	<0.001
Male	CVAI-D	0.58	0.54	0.62	8.74	55.47	58.09	0.14	0.91	<0.001
	LAP-D	0.60	0.56	0.64	52.30	74.52	42.74	0.17	0.91	<0.001
	TG/HDLC-D	0.53	0.49	0.57	1.63	89.95	18.67	0.19	0.90	0.146
	TYG-D	0.68	0.64	0.71	0.14	61.02	64.73	0.17	0.93	<0.001
Overall	CVAI-D	0.55	0.52	0.57	13.06	61.61	47.02	0.13	0.90	<0.001
	LAP-D	0.59	0.56	0.62	67.07	75.22	42.06	0.17	0.91	<0.001
	TG/HDLC-D	0.53	0.50	0.56	1.62	89.03	20.83	0.19	0.90	0.066
	TYG-D	0.66	0.63	0.69	0.13	58.14	66.87	0.16	0.93	<0.001

IQR, interquartile range; CVAI-D, difference in Chinese visceral adiposity index; LAP-D, difference in lipid accumulation product; TG/HDLC-D, difference in triglyceride/high-density lipoprotein cholesterol ratio; TyG-D, difference in triglycerides/ glucose index.

### Comparison of AUC values between TyG-D and other indexes for T2DM by gender

Table 4 shows the differences in AUC values between TyG-D and other indexes for T2DM. We found that differences in AUC values between TyG-D and other three indexes were significant both in male and female (all *P* < 0.001). Results showed that TyG-D had the strongest predictive performance for T2DM compared with other three surrogate indexes.

**Table 4:** Comparison of AUC values between TyG-D and other indexes by genders.

	**Difference between area**	***P*-value**
	**(95%CI)**	
**Female**
TyG-D vs. CVAI-D	0.134 (0.097, 0.171)	<0.001
TyG-D vs. LAP-D	0.061 (0.033, 0.088)	<0.001
TyG-D vs. TG/HDLC-D	0.122 (0.095, 0.148)	<0.001
**Male**
TyG-D vs. CVAI-D	0.097 (0.053, 0.142)	<0.001
TyG-D vs. LAP-D	0.080 (0.046, 0.114)	<0.001
TyG-D vs. TG/HDLC-D	0.145 (0.117, 0.174)	<0.001
**Overall**
TyG-D vs. CVAI-D	0.114 (0.084, 0.143)	<0.001
TyG-D vs. LAP-D	0.073 (0.051, 0.094)	<0.001
TyG-D vs. TG/HDLC-D	0.133 (0.114, 0.153)	<0.001

## Discussion

In this prospective cohort study, we analyzed the baseline and follow-up data of 4,596 Chinese participants and found that longitudinal changes in the four surrogate IR indexes were significantly associated with the risk of T2DM. TyG-D showed the highest predictive accuracy of all the indexes.

Although relevant studies have been conducted previously, they have some limitations. Li et al. found the association between the TyG and new-onset diabetes was positive and linear, with an AUC of 0.597 (0.559–0.636) (16). Compared to Li's study, our research found that the AUC of the TyG-D was 0.66 (0.63–0.69), which was higher than the TyG index alone in the risk of incident diabetes. It was possible that TyG-D had higher predictive performance than TyG index alone for the risk of T2DM. Zhang et al. reported that the risk of T2DM increased with elevated TyG index in normal-weighted Chinese and suggested that TyG might be an important indicator in identifying population at high risk of T2DM. However, the applicability and utility of TyG index for predicting T2DM in normal-weighted people should be further confirmed in the entire population (24).

In Chinese adults, IR showed a stronger correlation with T2DM risk compared to β-cell dysfunction (25). In clinical practice, in addition to BMI, measurement of WC might be useful in identifying and managing overweight/obese population at high cardiometabolic risk (26). Anthropometric parameters (WC, waist-to-hip ratio, BMI), surrogate measurements of IR (fasting plasma glucose, insulin, fasting insulin-glucose product), fasting lipids, SBP and DBP were also important in predicting T2DM risk (27). Although BMI was a major risk factor of T2DM-related mortality, many people with normal BMI were referred to as “metabolically obese but normal weight (MONW),” which was characterized by a variety of metabolic risk factors and would significantly increased T2DM risk in many ethnic groups.

TyG, a combination of fasting blood glucose and TG, was considered a surrogate marker of IR. Previous studies on middle-aged and elderly adults in China suggested that TyG could identify metabolic syndrome (Mets) (28, 29). There was a significant correlation between the TyG index and incidence of T2DM in individuals with obesity (including visceral fat obesity), fatty liver and normal BMI, which was not a measure of body fat distribution (30). In Japanese with normal glycemic levels, the longitudinal cohort research showed a positive correlation between baseline TyG-BMI and the risk of T2DM, which was noticeably higher in young people, women, non-hypertensive people, and non-drinkers (31). Previous studies suggested that TyG-waist-to-height ratio and TyG-BMI were both clinically effective markers for predicting diabetes in individuals with normal and impaired fasting glucose levels, respectively (16). Numerous studies on the TyG index in individuals of normal weight demonstrated the significance of the TyG index (23, 32).

For female, TyG, VAI and LAP indexes were considered better predictors for T2DM than conventional anthropometric and laboratory measures (27). Based on BMI, WC, TG, and HDL-C, VAI was a gender-specific indicator of visceral adiposity. The risk of new-onset T2DM was significantly correlated with baseline VAI and its transitions. Early prevention was required to reduce the risk of T2DM in Chinese with high VAI levels (33). LAP index, a phenotype of obesity produced by WC and fasting TG, was independently linked to T2DM in hypertensive female. Elevated LAP levels and patterns of low-to-high and maintained-high LAP transitions were significant risk factors for T2DM in women (34). Prevention was needed to combat T2DM at an early dyslipidemic stage. Previous studies suggested that TG/HDL-C was a better marker for assessing IR and DM in elderly Chinese when compared with other common lipid measurements (35, 36). For African American women, the TG/HDL-C ratio was a predictor of β-cell function (37), but it was not a predictor of IR (38). Although the TyG presented significantly better predictive performance than CVAI, LAP and TG/HDL-C in this study, it still required further research in other populations.

The data of this study were obtained from a cohort, so there was a strong causal inference. Compared to the traditional HOMA-IR detection method, surrogate IR indexes were cheaper, faster, and helpful to earlier prevention of T2DM. Researchers could fully utilize both the investigated data and laboratory data when considering the longitudinal changes of IR to reflect the dynamic changes of the human body. Calculating the difference of indexes could reduce the absolute difference between the data and avoid the influence of individual extreme values. This study provided a potential direction for predicting the risk of T2DM development. There were some limitations. First, the follow-up period of this study was not long enough. Second, as the majority of the study's participants were middle-aged and elderly individuals, further researches on other age groups would be necessary. Previous studies suggested that there was a slight difference in DM incidence between women and men. More researches should be encouraged to explore specific T2DM predictors in both women and men. Finally, we were unable to include some other potential confounders such as physical activity, diet and genes in our analysis, which were not contained in the database.

## Conclusion

Our study found that TyG-D index was a stronger predictor in the clinical setting for identifying individuals with T2DM in middle-aged and elderly Chinese compared with other indexes. Among the four analyzed indexes, TyG-D presented the largest AUC value [0.66 (0.63–0.69)] and highest sensitivity.

## Data availability statement

Publicly available datasets were analyzed in this study. This data can be found at: China Health and Retirement Longitudinal Survey (2020). Available online at: http://charls.pku.edu.cn/pages/data/111/zh-cn.html.

## Author contributions

YH and LP designed the study. LL, HP, JL, HW, and GX contributed to data acquisition. MW and LP performed the statistical analysis. JH and YH contributed to the discussion. LP and YG drafted the manuscript. JH edited the manuscript. All authors read and approved the final manuscript.

## Funding

This work was supported by the Research Program of Deyang Municipal Science and Technology Department (2022SCZ089 and 2022SCZ130).

## Conflict of interest

The authors declare that the research was conducted in the absence of any commercial or financial relationships that could be construed as a potential conflict of interest.

## Publisher's note

All claims expressed in this article are solely those of the authors and do not necessarily represent those of their affiliated organizations, or those of the publisher, the editors and the reviewers. Any product that may be evaluated in this article, or claim that may be made by its manufacturer, is not guaranteed or endorsed by the publisher.

## Abbreviations

DM: Diabetes mellitus
T2DM: Type 2 diabetes mellitus
CVAI: Chinese visceral adiposity index
LAP: Lipid accumulation product
TyG: Triglyceride-glucose
TG: Triglyceride
HDL-C: High-density lipoprotein cholesterol
BMI: Body mass index
WC: Waist circumference
DBP: Diastolic blood pressure
SBP: Systolic blood pressure.
